# Pattern of Depression Among Patients of Myocardial Infarction in Karachi, Pakistan: A Cross-sectional Study

**DOI:** 10.7759/cureus.3199

**Published:** 2018-08-24

**Authors:** Hasham Saeed, Fahad Khan, Syeda Fatima Mohsin, Farwa Haider Qizilbash, Tayyab Raza Fraz, Qirat Jawed, Nawaz Lashari

**Affiliations:** 1 Internal Medicine, Dow Medical College and Civil Hospital, Karachi, PAK; 2 Cardiology, Civil Hospital Karachi, Karachi, PAK; 3 Student, Dow Medical College and Civil Hospital, Karachi, PAK; 4 Statistics, University of Karachi, Karachi, PAK; 5 Cardiology, Civil Hospital, Karachi, PAK

**Keywords:** acute coronary syndrome, acs, depression, outcome, complication, myocardial infarction, mi, smoking

## Abstract

Background

Depression is a well-known risk factor that aggravates the chances of having various complications of acute coronary syndrome (ACS) such as cardiovascular collapse, heart failure, arrhythmia, recurrent myocardial infarction, and stroke among patients of ACS. ACS is a broad term which includes unstable angina as well as myocardial infarction (MI). The purpose of this study is to analyze the prevalence of depression among the patients of MI admitted to the tertiary care hospitals of Karachi, Pakistan.

Methods and materials

A hospital-based, cross-sectional study was conducted in which 375 admitted and diagnosed patients of MI with a mean age of 58 years were interviewed at the cardiology department of the Civil Hospital and National Institute of Cardio-Vascular Diseases (NICVD) Hospital, Karachi, from June to November 2017 using a self-made validated questionnaire, including patient health questionnaire-9 (PHQ-9).

Results

Overall, about 12.8% of the cases were screened positive for severe depression, 17.1% for moderately severe depression, 17.6% for moderate depression, and 32% for mild depression (total of 79.5%). Of 146 female subjects, 119 (81.5%) were found to be suffering from some degree of depression while 179 (78.2%) of the 229 males screened positive for some degree of depression. Furthermore, 79 (82.3%) of the 96 smokers were suffering from a range of depression while 219 (78.5%) of the 279 non-smokers suffered the same. In addition, the results of the PHQ-9 were cross-tabbed with age (p=0.34), gender (p=0.66), marital status (p=0.07), living status, smoking (p= 0.72), hypertension (p=0.55), and diabetes (p=0.19).

Conclusion

This study concludes that many of the patients of MI who were admitted to the tertiary care hospitals in Karachi, Pakistan, are suffering from major depressive behavioral changes following the cardiovascular event, which is known to aggravate the chances of having complications associated with it.

## Introduction

It is quite commonly observed that after a myocardial infarction (MI), cardiac surgery or procedure, recent hospitalization, or new diagnosis of heart disease, patients tend to get sad or depressed [1]. Temporary feelings of sadness are normal and should gradually go away within a few weeks. Sometimes, however, a depressed mood can prevent a patient from leading a normal life. When a depressed mood is severe and accompanied by other symptoms that persist every day for two or more weeks, it can deteriorate the patient’s condition and aggravate the damage, increasing the risk of worsening the prognosis of acute coronary syndrome (ACS) by a factor of 1.6 to 2.7-fold [2]; it can cause a 22% increased risk of all-cause mortality and a 13% increased risk of cardiovascular events [3]. This is due to several effects of unmanaged stress and depression like high blood pressure, arterial damage [4], irregular heart rhythms, weakened immune system, increased platelet activity [5], decreased heart rate variability, increased proinflammatory markers and cytokines [6-8] and negative lifestyle habits associated with depression (e.g., smoking, alcohol consumption, lack of exercise, poor diet, and isolation) [9-17]. Previous studies were conducted to analyze the prevalence of depression, post-MI, in developed countries and showed a dose-response relationship, with the severity of depression being associated with an earlier onset of complications [18-19].

A recent study based on the National Health Interview Survey (NHIS) data of 30,801 adults found the 12-month prevalence of major depression to be 9.3% in individuals with cardiac disease as compared with 4.8% in those with no comorbid medical illness [20]. Depression has received formal recognition as a risk factor for poor prognosis in patients with ACS [1-2]. There is a lack of knowledge regarding the above-described relationships in developing countries. This study was conducted to look for the prevalence of depressive behavioral changes in the ACS patients of two tertiary care hospitals of Karachi, Pakistan. Illiteracy and a lack of awareness about aggravating factors, leading to a poor prognosis for ACS patients in developing countries, makes it difficult to get to ensure increased morbidity and mortality in such patients.

## Materials and methods

**Figure 1:**
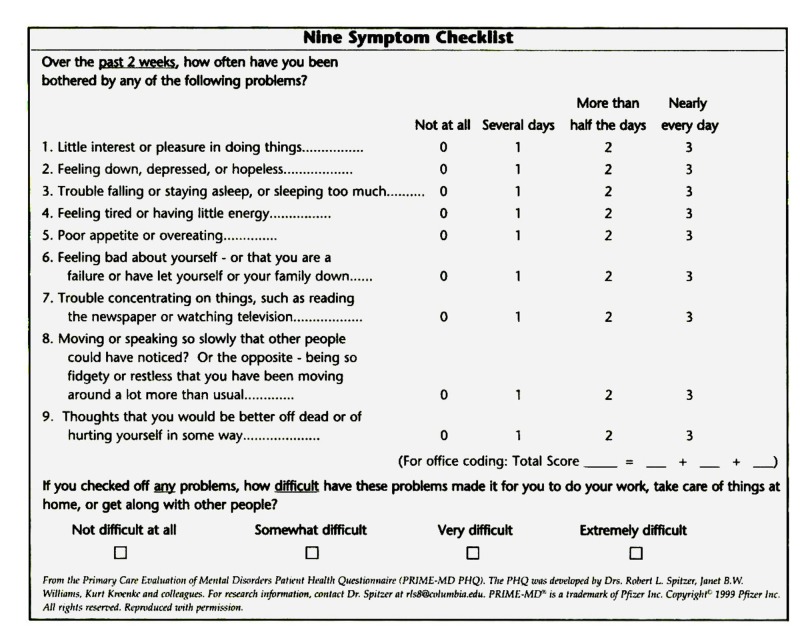
Patient health questionnaire-9 (PHQ-9)

A hospital-based, cross-sectional study was conducted at two sites, namely the cardiology departments of the Civil Hospital and National Institute of Cardio-Vascular Diseases (NICVD) over a period of three months from June to November 2017. The sample size was found to be 375 for the population survey, taking a 95% confidence interval using OpenEpi data version 3.03 (Emory University, Rollins School of Public Health, Atlanta, Georgia) [21]. The questionnaire included the following three sections, i.e., demographics, past medical, drug and family history and depression screening scale. The demographics section included age, gender, marital status, education, and living alone or not. In the history section, patients were asked about their smoking habits, whether they were diagnosed cases of hypertension and/or diabetes, and their family history of diseases. For the last section of depression screening, patient health questionnaire-9 (PHQ-9) scale was incorporated (Figure 1).

The questionnaire was asked through a standard protocol and was finalized after performing a pilot study on 25 cases. Patients with mentally or physically unstable conditions, and those with other life-threatening conditions and/or with valvular or septal defects, were excluded from our study while only those with an MI within three months with or without comorbidities (hypertension, diabetes, etc.) were included. None of the patients were on antidepressant treatment. Most of the subjects interviewed were illiterate (62.7%) or had only completed primary education (19%) and were unaware of the ways to avoid depression following MI.

Categorical data were presented as frequency and percentages while continuous data was presented in terms of mean and standard deviations. Statistical Package for the Social Sciences (SPSS) version 21 (IBM SPSS, Armonk, New York) was used for analyzing the collected data, and the chi square was then applied to determine the correlation between categorical variables. Patients with a PHQ-9 score of 1-4 were said to have screened negative for depression while those with scores of 5-9, 10-14, 15-19, and 20-27 were said to be suffering from mild, moderate, moderately severe, and severe depression, respectively.

## Results

The total number of individuals that participated in the study were 375. Table 1 depicts their demographic characteristics which include age, gender, marital status, education, and whether they live alone or not.

**Table 1:** Demographics

Characteristics	Frequency	Percentage
Age		
Mean (SD): 58.18 (10.67)		
37-50	105	28
51-58	90	24
59-65	92	24.5
66-90	88	23.5
Gender		
Male	229	61
Female	146	39
Marital status		
Single	24	6.4
Married	278	74.1
Divorced	12	3.2
Widow	61	16.3
Education		
Illiterate (no education)	235	62.7
Primary	71	18.9
Secondary	46	12.3
Graduation	13	3.5
Masters	10	2.7
Live alone		
Yes	22	5.9
No	353	94.1

Subjects were asked about their smoking habits, comorbidities (mainly hypertension and diabetes), and family history of diseases. The results of the responses are shown in Table 2.

**Table 2:** Other questions

Characteristics	Frequency	Valid Percentage
Smoking habits		
Yes	96	25.6
Packs per day?		
0.1-0.9	14	14.6
1.0-1.4	52	54.2
1.5-1.9	13	13.5
2.0-2.4	17	17.7
For how many years?		
1-19 years	35	36.5
20-29 years	28	29.2
30-55 years	33	34.4
No	279	74.4
Diagnosed hypertension		
Yes	324	86.4
No	51	13.6
Diagnosed diabetes mellitus		
Yes	160	42.7
No	215	57.3
Family history		
Yes	261	69.6
Hypertension	107	41
Diabetes mellitus	28	10.7
Coronary artery disease (CAD)	56	21.5
No	114	30.4

**Figure 2:**
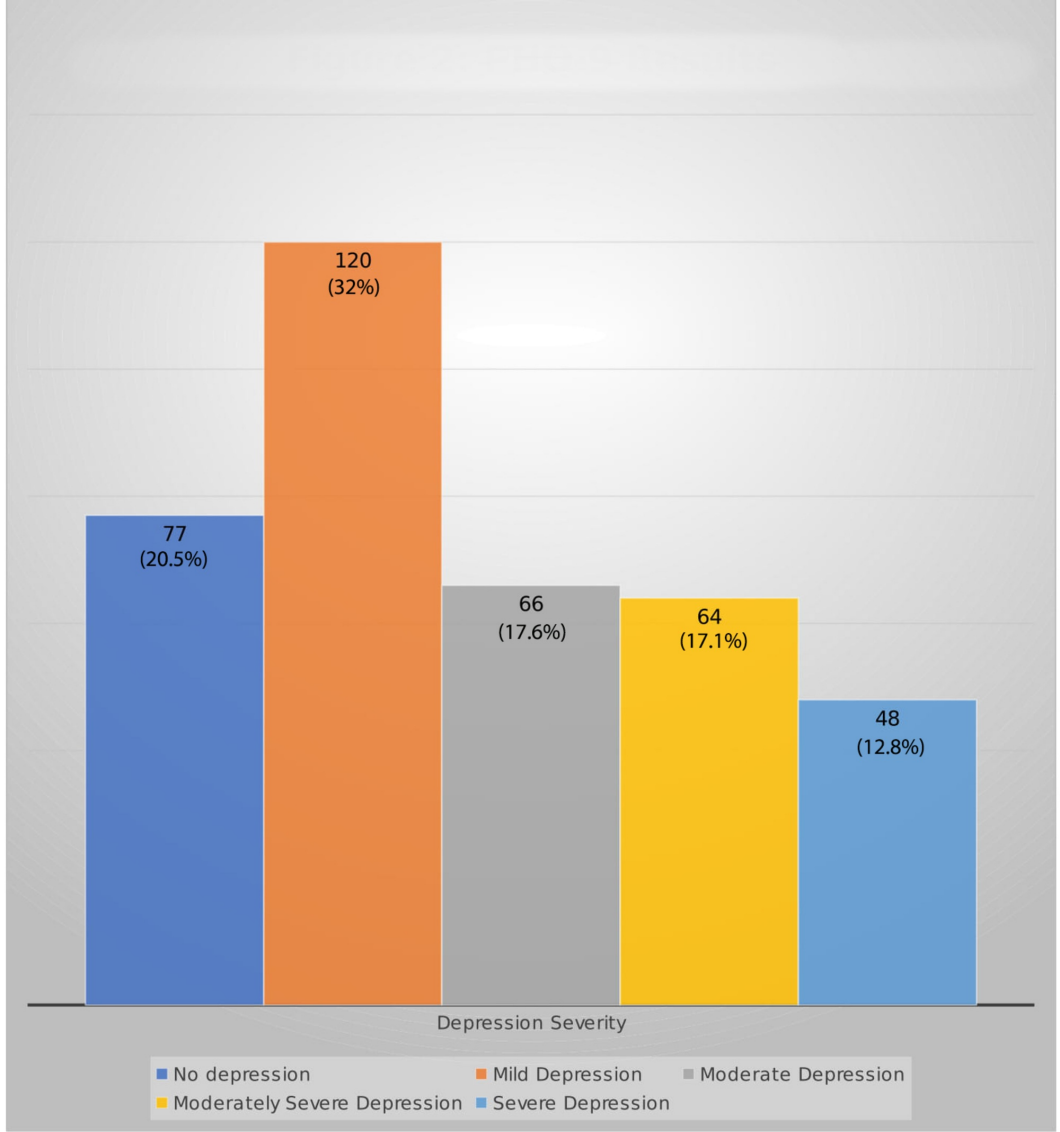
Patient health questionnaire-9 (PHQ-9) results

Results of the PHQ-9 questionnaire are shown in Figure 2.

The results of the PHQ-9 were cross-tabbed with age, gender, marital status, living status, smoking, hypertension, and diabetes (Table 3).

**Table 3:** Cross tabulation

	No Depression n (%)	Mild Depression n (%)	Moderate Depression n (%)	Moderately Severe Depression n (%)	Severe Depression n (%)	Chi-Square (p-value)	Total n
Age						13.38 (0.34)	
37-50	21 (20)	42 (40)	19 (18.1)	14 (13.3)	9 (8.6)		105
51-58	21 (23.3)	20 (22.2)	13 (14.4)	20 (22.2)	16 (17.8)		90
59-65	18 (19.6)	31 (33.7)	16 (17.4)	13 (14.1)	14 (15.2)		92
66-90	17 (19.3)	27 (30.7)	18 (20.5)	17 (19.3)	9 (10.2)		88
Gender						2.36 (0.66)	
Male	50 (21.8)	67 (29.3)	40 (17.5)	41 (17.9)	31 (13.5)		229
Female	27 (18.5)	53 (36.3)	26 (17.8)	23 (15.8)	17 (11.6)		146
Marital status						19.61 (0.07)	
Single	5 (20.8)	14 (58.3)	3 (12.5)	2 (8.3)	0		24
Married	61 (21.9)	81 (29.1)	47 (16.9)	50 (18)	39 (14)		278
Divorced	3 (25)	4 (33.3)	0	2 (16.7)	3 (25)		12
Widow	8 (13.1)	21 (34.4)	16 (26.2)	10 (16.4)	6 (9.8)		61
Live alone						N/A	
Yes	8 (36.4)	7 (31.8)	3 (13.6)	2 (9.1)	2 (9.1)		22
No	69 (19.5)	113 (32)	63 (17.8)	62 (17.6)	46 (13)		353
Smoking						2.08 (0.72)	
Yes	17 (17.7)	29 (30.2)	16 (16.7)	20 (20.8)	14 (14.6)		96
No	60 (21.5)	91 (32.6)	50 (17.9)	44 (15.8)	34 (12.2)		279
Hypertension						3.03 (0.55)	
Yes	66 (20.4)	108 (33.3)	55 (17)	56 (17.3)	39 (12)		324
No	11 (21.6)	12 (23.5)	11 (21.6)	8 (15.7)	9 (17.6)		51
Diabetes mellitus						6.05 (0.19)	
Yes	41 (25.6)	50 (31.3)	22 (13.8)	26 (16.3)	21 (13.1)		160
No	36 (16.7)	70 (32.6)	44 (20.5)	38 (17.7)	27 (12.6)		215

Out of the total 146 female subjects, 119 (81.5%) were found to be suffering from some degree of depression. On the other hand, 179 (78.2%) of the 229 males screened positive for some degree of depression. Furthermore, 79 (82.3%) of the 96 smokers were suffering from a range of depression while 219 (78.5%) of the 279 non-smokers suffered the same.

## Discussion

In past studies, approximately 20% of the patients hospitalized for ACS met the American Psychiatric Association’s Diagnostic and Statistical Manual of Mental Disorders (DSM) criteria for major depression, and an even larger percentage showed subclinical levels of depressive symptoms [22]. In our study, approximately 30% of the subjects were suffering from moderately severe to severe depression.

In patients with coronary heart disease, there is also an association between depression and functional impairment [23], which suggests that depression may exacerbate physical inactivity and poor self-care. This might be a factor in our study as well. However, it is important to recognize that any candidate's mechanism is not mutually exclusive, and multiple potential mechanisms may link depression with adverse outcomes. For instance, a study revealed a significant association between smoking and depressive symptoms [24], but our findings failed to do so.

There is some evidence that depressive episodes that develop soon after an MI may carry a higher risk than episodes that begin before MI. In a study, it was reported that a subgroup of patients who were depressed and/or anxious after the MI and who had denied ever being depressed before the acute event were at increased risk of cardiovascular mortality during the follow-up period [25-26]. Therefore, it is crucial to screen every ACS patient for depression to avoid a delay in the diagnosis and treatment of depression. A recent randomized, controlled trial revealed that a 24-week treatment of depression following recent ACS lowers the risk of major adverse cardiac events after a median of 8.1 years, thereby improving long-term cardiac outcomes [27].

## Conclusions

It is proven from previous studies that treating depression improves long-term survival after MI and that worsening depression increases the risk of adverse clinical outcomes. Moreover, severe or persistent depression is reason enough to consider more comprehensive evaluation and treatment. Further research is needed to determine the risks and benefits of routine screening for depression and to identify safe and effective treatment options for depression in patients with MI.
